# Systematic Review of the Diagnostic and Clinical Utility of Salivary microRNAs in Traumatic Brain Injury (TBI)

**DOI:** 10.3390/ijms232113160

**Published:** 2022-10-29

**Authors:** Matthew I. Hiskens, Tesfaye S. Mengistu, Katy M. Li, Andrew S. Fenning

**Affiliations:** 1 Mackay Institute of Research and Innovation, Mackay Hospital and Health Service, 475 Bridge Road, Mackay, QLD 4740, Australia; 2 School of Health, Medical and Applied Sciences, Central Queensland University, Bruce Highway, Rockhampton, QLD 4702, Australia; 3 Faculty of Medicine, School of Public Health, University of Queensland, 266 Herston Road, Herston, QLD 4006, Australia

**Keywords:** biomarker, saliva, diagnosis, concussion, post-concussion syndrome

## Abstract

Research in traumatic brain injury (TBI) is an urgent priority, as there are currently no TBI biomarkers to assess the severity of injury, to predict outcomes, and to monitor recovery. Small non-coding RNAs (sncRNAs) including microRNAs can be measured in saliva following TBI and have been investigated as potential diagnostic markers. The aim of this systematic review was to investigate the diagnostic or prognostic ability of microRNAs extracted from saliva in human subjects. PubMed, Embase, Scopus, PsycINFO and Web of Science were searched for studies that examined the association of saliva microRNAs in TBI. Original studies of any design involving diagnostic capacity of salivary microRNAs for TBI were selected for data extraction. Nine studies met inclusion criteria, with a heterogeneous population involving athletes and hospital patients, children and adults. The studies identified a total of 188 differentially expressed microRNAs, with 30 detected in multiple studies. MicroRNAs in multiple studies involved expression change bidirectionality. The study design and methods involved significant heterogeneity that precluded meta-analysis. Early data indicates salivary microRNAs may assist with TBI diagnosis. Further research with consistent methods and larger patient populations is required to evaluate the diagnostic and prognostic potential of saliva microRNAs.

## 1. Introduction

Traumatic brain injury (TBI) is a global health issue [1,2] with an estimated sixty-nine million individuals worldwide sustaining a TBI each year [3]. TBI is defined as the disruption in brain function, or other evidence of brain pathology, caused by an external physical force [4]. TBI is a significant contributor to morbidity, mortality, and economic burden [5,6,7,8]. Research in TBI is an urgent priority, as there are currently no biomarkers to assess the severity of injury, to predict outcomes, and to monitor recovery. A TBI biomarker would also serve as a measure of therapeutic efficacy in the development of a TBI treatment, which is of importance as all pharmacological agents that have been tested in Phase III clinical trials have failed [9].

Currently, the diagnosis of TBI is time consuming, expensive, and often inconclusive. Current clinical guidelines involve a neurological assessment including Glasgow Coma Scale (GCS) grading of the severity of brain injury. For suspected head injury in sport, the Sport Concussion Assessment Tool 5th edition (SCAT5) is used for acute evaluation [10]. However, these tools may be imprecise due to non-specific signs, transient and subjective symptoms, and diverse diagnostic criteria [11,12], which may contribute to under treatment [13]. In addition to neurological assessment, structural neuroimaging may be undertaken, most commonly via computed tomography (CT) and magnetic resonance imaging (MRI) [4]. However, CT scans may not provide clear and objective information for diagnosis, especially in mild TBI where CT is negative by definition [14] More sophisticated neuroimaging techniques provide improved functional and microstructural imaging capabilities; however, these methods may be impractical for use in clinical settings and are not dynamic enough to be useful for rapid analysis during athletic settings [15]. Therefore, an explicit and objective TBI biomarker is required to aid clinical diagnosis and for use in patient care. 

Fluid biomarkers represent an attractive strategy for providing diagnostic and prognostic accuracy in TBI [4,16]. The search for a reliable biomarker to assess the severity of TBI, to predict recovery, and to serve as a measure of therapeutic efficacy in clinical trials has identified several options in cerebrospinal fluid (CSF) and blood. The protein biomarkers S−100 B, GFAP, NSE, NF-L, tau, and UCH-L1 have been investigated as blood biomarkers of TBI. However, these proteins have low concentrations in blood due to biological limitations involving inability to cross the blood–brain barrier (BBB), clearance via the liver or kidneys, and carrier protein binding, and as a result are limited due to low sensitivity and specificity [17,18]. CSF sampling has limited applicability in cases of mild TBI, which make up 75% of Emergency Department cases [19]. Additionally, blood and CSF biomarkers have limited practicality outside of clinical settings [16,20], and may not be well-utilized in mild TBI where blood samples are not routinely taken, are time consuming, and reducing the placement of unnecessary cannulas is an ongoing initiative [21]. Therefore, the most well-researched approaches in blood and CSF TBI biomarker development may have inherent challenges and limited applicability for use. 

Recent advances in high-throughput technology, including next generation sequencing (NGS), have identified new classes of RNA species as candidate biomarkers. Among these, microRNAs (miRNAs) are a class of small non-coding RNAs (sncRNA) that are involved in post-transcription regulation of molecular function involving mRNA degradation and mediation of protein synthesis [22,23]. miRNAs control diverse biological processes involving cell metabolism and regulation [24], with individual miRNAs able to regulate hundreds of target mRNAs [23,25]. Based on these properties, miRNAs are emerging as candidate biomarkers in the diagnosis, prognosis, and treatment of TBI [2,23,26]. Compared to protein-based biomarkers, miRNAs may achieve superior sensitivity due to stability in peripheral fluids, ability to cross the BBB, and protection by RNA-binding proteins and microvesicles such as exosomes [27]. Several studies have identified the potential of microRNAs as blood-based TBI biomarkers [23,25,28,29].

Recently, in addition to peripheral blood-based miRNAs, salivary miRNAs have shown potential in the diagnosis and prognosis of TBI. Therefore, the aim of this systematic review and meta-analysis was to systematically investigate the diagnostic or prognostic ability of microRNA biomarkers extracted from saliva in human TBI subjects.

## 2. Methods

### 2.1. Search Strategy

We conducted the systematic search in PubMed, Embase, Scopus, PsycINFO and Web of Science using different combinations of key terms and medical subject heading [MeSH Terms] with relevant filters (Supplementary Table S1). We used Boolean operators (“AND” or “OR”) to combine key words or independent searches. We also used truncations to capture words that could have multiple endings. PubMed search strategy is presented as an example: ((((“microrna”[tiab] OR “mirna”[tiab] OR “mirnas”[tiab] OR “micro rna”[tiab] OR “micrornas”[tiab] OR “biomarker*”[tiab] OR “saliva”[tiab]) AND “diagnos*”[tiab]) OR “prognos*”[tiab] OR “predict*”[tiab]) AND (“brain injuries, traumatic”[MeSH Terms] OR (“brain”[All Fields] AND “injuries”[All Fields] AND “traumatic”[All Fields]) OR “traumatic brain injuries”[All Fields] OR (“traumatic”[All Fields] AND “brain”[All Fields] AND “injury”[All Fields]) OR “traumatic brain injury”[All Fields] OR “traumatic brain injury”[tiab] OR “TBI”[tiab] OR “traumatic brain injuries”[tiab] OR “brain trauma”[tiab] OR “cerebral trauma”[tiab] OR “cerebrovascular trauma”[tiab] OR “encephalopathy traumatic”[tiab] OR “mild traumatic brain injury”[tiab] OR “posttraumatic encephalopathy”[tiab] OR “traumatic brain injuries”[tiab] OR “traumatic brain lesion”[tiab] OR “traumatic cerebral lesion”[tiab] OR “traumatic encephalopathy”[tiab] OR “CTE”[tiab] OR “chronic traumatic encephalopathy”[tiab])) AND ((ffrft[Filter]) AND (clinicalstudy[Filter] OR clinicaltrial[Filter] OR comparativestudy[Filter] OR controlledclinicaltrial[Filter] OR multicenterstudy[Filter] OR observationalstudy[Filter] OR randomizedcontrolledtrial[Filter] OR validationstudy[Filter]) AND (humans[Filter]) AND (english[Filter])). Reference lists from identified articles and review articles were also searched. The electronic database search was conducted from 14–17 February 2022 without publication year restrictions.

### 2.2. Study Selection and Exclusion Criteria

Citations retrieved from all electronic databases were imported to EndNote x9 and duplicates were removed. Two reviewers (TSM and MH) independently performed title and abstract screening using the Population/participants, Interventions, Comparisons, Outcomes, and Study design (PICOS) framework [30]. Full-text screening was conducted in the second round to decide studies’ eligibility. Disagreements in screening and study selection processes were resolved through discussion. 

We included only studies conducted on human populations and published in English meeting the following criteria: (i) original studies of any design, and (ii) articles presented diagnostic or prognostic capacity of salivary microRNAs for TBI. We excluded studies: published in non-English language, animal or cell experiment studies, systematic reviews, case series/reports, conference papers/abstracts, proceedings, editorial reviews, letters of communication, commentaries, and qualitative studies.

### 2.3. Data Extraction and Analysis

Two reviewers (TSM and MH) checked the lists of selected articles. TSM extracted data on author’s name, publication year, country, study type/design, study participants, study sample, age and sex, source of microRNA, microRNA expression (type), microRNA detection technique, TBI severity and diagnostic/prognostic performance indicators. MH checked extraction completeness, accuracy, and quality of extracted data. Data were thematically synthesized and narrated. Findings are reported following the Preferred Reporting Items for Systematic Reviews and Meta-Analyses (PRISMA) guidelines [31].

## 3. Results

### 3.1. Study Selection and Characteristics

**Figure 1:**
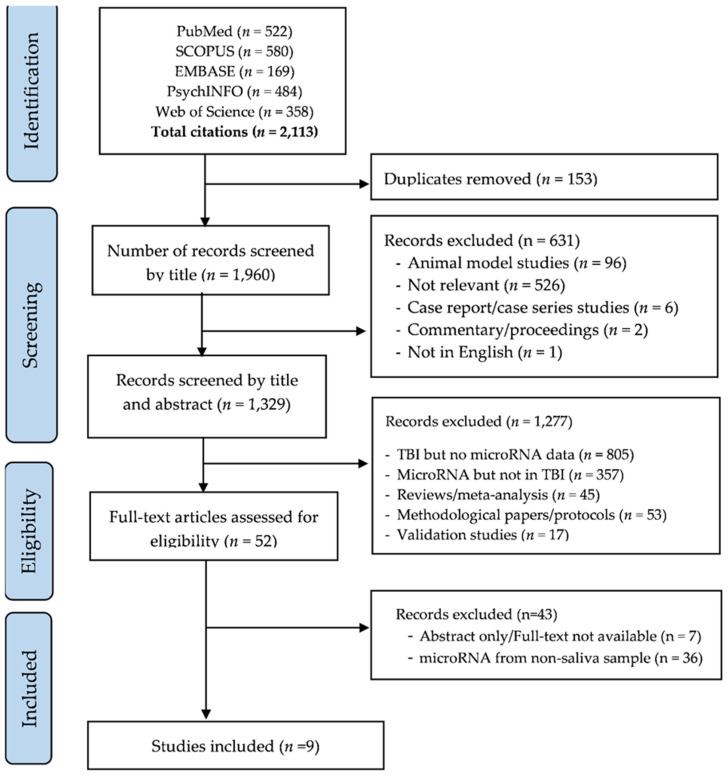
PRISMA flow diagram of screening and selection process.

Figure 1 shows the process of study screening, selection, and reasons for exclusions. The initial search yielded 2113 candidate studies. We screened studies by title, excluded duplicates (*n* = 153) and studies that did not meet inclusion criteria (*n* = 631). Of 1329 studies screened by abstract, 1277 were excluded for indicated reasons and 52 studies were selected for full-text review. A total of 9 studies [32,33,34,35,36,37,38,39,40] were eligible for inclusion (Figure 1). 

The characteristics and key findings of the included studies are summarised in Table 1, including the sex and age composition of populations, microRNAs extraction time, identified microRNAs and key findings. All the included studies were conducted in the UK [32,33] and USA [34,35,36,37,38,39,40]. Of the nine included studies [32,33,34,35,36,37,38,39,40], four were case–control studies [33,34,37,38], three were cohort studies [32,39,40], one study used both case–control and cross-sectional study designs [36] and one study used case–cohort design [35]. Eight studies [32,33,34,36,37,38,39,40] involved subjects who had only mild TBI, with one study including a cohort of severe TBI [35]. Studies collected initial samples from as early as 30 min post-activity [40] up to 14 days following injury [35,38,39]. In all the studies more than 50% of the subjects were male. Seven studies had control groups that had not experienced a TBI [32,33,35,36,37,38,40], and the remaining two studies assessed the prevalence of post-concussion syndrome (PCS) and used control groups with subjects that had an acute TBI with no PCS symptoms [34,39]. Five studies used saliva collection that involved expectoration into a collection container designed for sample preservation [32,33,35,37,39], while three studies used a sample collection sponge designed for this purpose [34,36,38], and one study used both methods [40].

### 3.2. Circulating miRNAs and Their Relative Expression

The miRNAs differentially expressed in the saliva of TBI patients varied greatly between studies. Overall, there were 188 miRNAs that were up- or down-regulated in the nine studies. Of these, 30 were reported in more than one study, and Table 2 summarizes these miRNAs identified in multiple studies and the direction of change between groups (upregulated or downregulated in TBI cases relative to healthy controls, or in PCS cases compared with those that did not have prolonged symptoms). 14 miRNAs were reported to be differentially expressed in a consistent direction across multiple studies.

## 4. Discussion

This systematic review assessed the utility of salivary miRNAs to diagnose TBI. We identified nine studies that met our inclusion criteria. While previous systematic reviews have reported TBI-related microRNAs from all biological sources [41], to the best of our knowledge, this is the first systematic review to perform a focused systematic analysis of miRNAs in saliva. In this study meta-analysis was not deemed possible due to the small number of studies and heterogeneity of design variables including saliva sample collection time, microRNA measurement techniques and variant microRNA expressions. The included studies also had variations in study size, study population, method of data pre-processing, and analysis method, which have been identified as challenges to perform meta-analysis in biomarker studies [42,43,44]. Thus, this systematic review summarised a wide variety of differentially expressed miRNAs in mTBI subjects qualitatively. 

Due to the diverse signaling responsibilities of miRNAs and the heterogeneity of TBI pathogenesis, it has been widely recognized that a panel of biomarkers will provide the greatest predicative performance in the diagnosis and prognosis of TBI [28]. Reflective of this approach, the studies of this review utilized miRNA panels including between five [33] and 21 [40] differentially expressed salivary miRNAs. These panels displayed varying diagnostic performance. Di Pietro et al. [32] identified a panel of 14 salivary miRNAs that predict mTBI immediately after injury with a 91% diagnostic performance, with predictive performance that increased to 96% 36–48 h after injury. In an earlier study, Di Pietro et al. [33] identified a panel of five differentially expressed miRNAs that had an AUC of 83.6% after 48–72 h from injury. Hicks et al. [35] identified a different panel of six miRNAs with similar (AUC of 85.2%) accuracy. Additionally, Hicks et al. [37] identified 11 miRNAs not confounded by exercise for prediction of sport related concussion (SRC). Of these 11 expressed miRNAs, the authors highlight that miR−27a−5p was the most accurate miRNA with AUC of 78%. 

The diagnostic performance of these studies is promising, as is the data in Table 2, which served to identify the most prevalent salivary miRNAs that appeared across multiple studies. Out of the 188 miRNAs identified in the nine studies, 30 appeared in multiple studies, and 14 of these had expression change direction that was consistent across studies [32,33,34,35,36,37,38,39,40]. For salivary miRNAs to be considered in clinical application they will need to demonstrate replicability from one study to the next with consistent direction of change, and from these 14 miRNAs may be candidates that hold the most promise for future application. Additionally, as more detail on the indications of specific microRNAs is understood, it may be that the inclusion of individual microRNAs could be combined with other markers to improve diagnostic accuracy, as is done in cancer literature [45].

In addition to correlating with a TBI diagnosis, several of these key microRNAs showing parallel expression have neurobiological pathways associated with TBI. Among these is let−7i−5p, which di Pietro and colleagues reported the best classifier of concussion in their initial study [33]. Let−7i is highly enriched in the brain and plays a role in the regulatory pathways of several neuroinflammatory modulators and cytokines [46]. In addition to a candidate for TBI biomarkers, let−7i has been implicated in Alzheimer’s disease and depression [32]. Let−7i upregulation was previously shown in CSF and serum in a rodent model of blast overpressure wave TBI, where it was involved in regulatory pathways of neuroinflammatory proteins [47]. Another promising marker is miR−27a−5p, which was downregulated in studies by Hicks and colleagues [36,37]. The miR−27a/b families are inhibitory factors of apoptosis [48] and regulate the sensitivity of neurons to apoptosis [49]. Additionally, miR−27a has been implicated in glutamate receptor signalling and GABA receptor signalling [28], protection from BBB disruption [50], TGFβ signalling [36], and diverse neuroinflammation processes [51]. Recently, Shultz and colleagues have identified decreased miR−27a−3p levels in the plasma of concussed football players six days after injury, which was inversely correlated with concussion symptom severity [52]. The Shultz study complements the existing literature in saliva and highlights the potential of miR−27a as a marker of TBI. Another miRNA with consistent expression change is miR−320c. Hicks and colleagues reported that miR320 c was downregulated in the saliva of mild TBI subjects and CSF in severe TBI patients with concentration in both fluids correlated with time since injury [35]. The study found a significant correlation between SCAT3 performance and attention difficulty. miR−320c expression was also reduced in the study by Johnson et al. where it showed a relationship with memory difficulty [39]. miR−320c has shown relation to pathways of plasticity regulation [53], and it has been implicated in pathways of severe depression, with altered levels found in the pre-frontal cortex of those who completed suicide [54]. While these are not the only microRNAs that may hold promise in a clinical setting, the current evidence involving consistent expression across a variety of biofluids and identified involvement in pathways of CNS injury and repair make these markers of interest.

As mentioned, there was variation in many of the findings of these studies, and there are key reasons for this. Many of these studies offer a cross-sectional indication of miRNA expression, with some studies undertaking initial sample collection at delayed time-points, meaning that the time-course of biomarker upregulation following mTBI is unclear. Indeed, a restriction in study comparison is the varied sample collection time relative to time of injury (ranging from <30 min to 14 days post-TBI). This broad sampling window will involve differing biological mechanisms of microRNAs and their role as molecular regulators. As molecular signatures undergo change in amplitude and direction following TBI, this disparity in study timeframes make comparisons difficult, and may mean that some markers that share direction of expression change were not identified. Many of the studies did not include repeat time-points, making it difficult to ascertain biomarker kinetics and the evolution of levels during the post-TBI phase. The 2021 study by Di Pietro et al. [32] did examine performance over multiple time points, and found that as time elapsed after injury there was increased expression of miRNAs in saliva [32]. Additionally, most studies focussed on acute change in biomarker expression, and did not extend to subacute timepoints, with the latest samples collected at 31–60 days post-TBI [38]. In conjunction with this, data involving long-term outcomes including return to work/play/activities of daily living and chronic symptoms of subjects was not collected. 

miRNA biomarker studies compare TBI subjects and healthy controls, but there are still many unknowns in the variables impacting the abundance of miRNAs. As such, the evaluation of miRNA biomarker variability in heterogeneous or comorbid populations is a challenge. For example, the studies of this review included a mix of male and female subjects, however sex-based differences may influence miRNA expression [55] including the regulation of gonadal hormones [56], menstrual cycles [57] and pregnancy [58]. The diet of subjects may also affect miRNA expression by contributing external miRNAs that are indistinguishable from endogenous miRNAs [59]. Circadian rhythms may also influence variations in miRNA levels, which was a factor that was only controlled in some studies [35]. Additionally, the findings of these studies may not be generalisable to standard TBI populations as many of these studies were undertaken in athletes. On one hand an athlete cohort may reduce confounding from chronic diseases and comorbidities. Conversely, this may introduce the role of exercise as a confounding variable in miRNA expression, as physical activity is known to affect circulating miRNA expression [60,61]. In young athletes performing an eight-week training program, plasma mir−93, miR−16, and miR−222 expression was altered compared with baseline [62]. However, it should be noted that none of these markers overlapped with those presented in Table 2 as potential pathologically expressed biomarkers. In ultramarathon athletes, miRNA expression change share pathways associated with cancer and inflammation, as well as BDNF signalling [63]. The type of exercise also influences miRNAs expression, with plasma miR−126 and miR−133 showing differing responses following marathon running, four hours of bicycle riding, and prolonged resistance training [64]. Hicks et al. attempted to identify salivary miRNAs unaffected by exercise, and further work and validation is required in finding a robust TBI biomarker independent of exercise [37]. Overall, there are many factors that require further investigation to ascertain their influence in miRNA expression.

Salivary miRNA biomarkers represent a distinct branch of biomarkers from other biofluids, and it is anticipated that there is significant variation in expression change from miRNAs derived from saliva compared with blood and CSF. Previous studies have shown that biofluid type can influence circulating miRNA expression and is a crucial factor underpinning inconsistent results, with miRNA expression differing between whole blood, serum and plasma samples [65]. This was demonstrated by Hicks and colleagues, who reported differences in direction of expression change for microRNAs in saliva compared with CSF [35]. In mixed martial arts fighters, the direction of expression change also showed differences in saliva and serum in more than half of the detected microRNAs [40]. Understanding the processes that allow peripheral migration of miRNAs from the CNS into the saliva following TBI will allow further understanding of this differentiation. While there is plausible evidence for serum biomarker expression that reflects underlying pathology via the disruption of the BBB following TBI, it is not clear how this pathology is transmitted to saliva. One hypothesis is the role of sensory (V, VII, IX) and motor (XII, X, XII) cranial nerves that may provide a route to communicate CNS damage [66]. Another potential route of microRNA transmission is via the glymphatic system, which assists in clearance of pathological CNS cells following TBI [67]. Future studies will be required to examine these mechanistic questions.

The identification of microRNAs involved in TBI signalling may have application beyond injury diagnosis, as microRNA involvement in physiological and pathological processes position them as targets to direct pharmacological therapy [68]. While a complete discussion on the therapeutic application of miRNA medication is beyond the scope of this review, it is worth illustrating the value of miRNA discovery for this purpose. miRNAs possess features which may allow their use in therapeutic design, including their ability to influence multiple targets through the manipulation of a single miRNA, and their short length of ~22 nucleotides [69]. miRNA-based therapeutics include miRNA mimics, which act as compensatory agents in situations of miRNA downregulation, and miRNA inhibitors which can be used for suppression of overexpression in relation to injury and disease [68]. The exploration of miRNAs as biomarkers of TBI injury will allow further development of effective therapeutics, in a condition where there is currently no treatment [70].

The current review had several limitations. The small number of papers meeting inclusion criteria is important to identify. Five databases were utilised, and articles were not limited by date, so it is unlikely that important evidence has been omitted. Considering that we included only nine papers exploring salivary miRNAs as TBI biomarkers, the conclusions that can be drawn presently are preliminary, and current evidence requires further investigation. While the heterogeneous study design has been previously highlighted, two studies included in the review investigated markers of PCS compared with non-PCS TBI, and as such did not include an uninjured control group. This may have had implications for the comparison of expressed miRNAs with other studies. Finally, it was not within the scope of this paper to describe the secondary injury mechanisms that influence the production of these miRNAs.

## 5. Conclusions

The initial findings identified in this systematic review highlight the potential of salivary miRNAs as TBI biomarkers. Currently, there are knowledge gaps in the practical application of these markers in relation to details such as marker kinetics and the influence of biological factors. As such, the current evidence is not suitably mature to identify specific microRNAs for application. Instead, future studies should narrow the sample collection time, investigate miRNA biomarker signatures across repeated time points, and reduce variation in study techniques and analysis methods.

## Figures and Tables

**Table 1:** Study characteristics.

Author (Year)	Study Type/Design	Study Participants	Study Sample (*n*)			Sex	Age Range or Mean (SD)	Saliva Sample Collection Time	microRNA Measurement	microRNA Expression (Type)	Summary of Key Findings	Reported AUC or ROC (95% CI)
			Total	Cases	Controls							
Di Pietro, V. et al. (2018) [33]	Case-control	Rugby players	12 (for discovery group)	6 Concussed (For discovery group)	6 non-concussed (For discovery group)	Male	16–65 years	48–72 hrs. from concussion	Nano-string Profiling	let-7i-5p, miR-142-3p, miR-107, miR-27b-3p, miR-135b-5p	- 5 differentially expressed miRNAs were identified with potential utility to distinguish concussed athletes from non-concussed athletes after 48–72 h from injury	AUCs for: - miR-27b-3p (0.755; 0.575–0.934) - let-7i-5p (0.845; 0.681–1) - miR-142-3p (0.791; 0.634–0.948) - miR107 (0.732; 0.565–0.904) - miR-135b-5p (0.755; 0.573–0.936) - Panel of 5 microRNAs (0.836; 0.669–0.995)
			32 (validation group)	22 concussed (validation group)	10 non-concussed (validation group)							
Di Pietro, V. et al. (2021) [32]	Prospective cohort	Rugby players	324	106 HIA+	HIA- non-concussed (*n* = 50); Uninjured (*n* = 102); musculoskeletal injury (*n* = 66)	Male	- HIA+ group 27.1 y - HIA- group 27.5 - Uninjured group 26.4 y - MSK group 25.9 y	3 sample collection time points for 2 seasons - In-game (T1) - Immediate post-game (T2) - Post-game (36–48 h)	NGS	32 differentially expressed from HIA+ and HIA- compared at T1, T2, T3 in season 1: let-7a-5p, miR-1246, let-7f-5p, let-7i-5p, miR-107, miR-148a-3p, miR-135b-5p, miR-126-3p, miR-21-5p, miR-465, miR-34b-3p, miR-92a-3p, miR-6, miR-476, miR-144-3p, miR-103a-3p, RNU6-6, RNU6-4, RNU6-45, RNU6-7, RNU6-73, tRNA27-MetCAT, tRNA18-ArgCCT, tRNA2-LeuTAA, Y_RNA.255, SNORD3B-2, tRNA120-AlaAGC, U2.3, snoU13.120, tRNA73-ArgCCG, U6.375, U6.601 14 with the highest accuracy compared with HIA- and controls combined as a panel in season 2: let-7a5p, miR-143-3p, miR-103a-3p, miR-34b-3p, RNU6-7, RNU6-45, Snora57, snoU13.120, tRNA18Arg-CCT, U6-168, U6-428, U6-1249, Uco22cjg1, YRNA_255	- 32 sncRNAs were differentially expressed across the two groups with let-7f-5p showing the highest AUC at 36–48 h - Panel of 14 salivary sncRNAs could differentiate concussion in cases and controls immediately after the game (AUC 0.91, 95% CI 0.81–1.00) and 36–48 h later (AUC 0.94, 95% CI 0.86–1.00) - Panel microRNA predictive performance to identify concussed players (AUC 0.96, 95% CI 0.92–1.00 post-game and AUC 0.93, 95% CI 0.86–1 at 36–48 h)	Season 1 **T1 AUC HIA+** **Vs.** HIA- =1.00 (1.00–1.00) **T2 AUC HIA+** Vs. All combined: 0.91 (0.81–1.00) Vs. HIA-: 0.88 (0.74–1.00) Vs. Uninjured: 0.93 (0.84–1.00) Vs. MSK: 0.90 (0.78–1.00) Vs. Baseline: 0.95 (0.90–1.00) **T3 AUC HIA+** Vs. All combined: 0.94 (0.86–1.00) Vs. HIA-: 0.96 (0.89–1.00) Vs. Uninjured: 0.96 (0.88–1.00) Vs. MSK: 0.90 (0.69–1.00) Vs. Baseline: 0.91 (0.84–0.98) Season 2 **T2 AUC HIA+** Vs. All combined: 0.96 (0.92–1.00) Vs. HIA−: 0.94 (0.85–1.00) Vs. Uninjured: 0.94 (0.87–1.00) Vs. MSK: 1.00 (1.00–1.00) **T3 AUC HIA+** Vs. All combined: 0.93 (0.86–1.00) Vs. HIA−: 0.86 (0.73–1.00) Vs. Uninjured: 0.95 (0.89–1.00) Vs. MSK: 0.95 (0.88–1.00)
Fedorchak, G. et al. (2021) [34]	Case-control	Patients with clinical diagnosis of mTBI	- 112 patients - 505 saliva samples	- 32 PPCS	- 80 non-PPCS	- 49 F - 63 M	8–24 y	2 time point sample collection - Initial (≤14 days post-injury) - Follow-up (≥21 days post injury)	NGS	wiRNA_48, miR-1246, miR-486-59, wiRNA_147, wiRNA_1590, wiRNA9924, miR-92b-3p, wiRNA_7971, miR-203a-5p, SNORD81, wiRNA_9447, miR-148a-5p, wiRNA_1385, wiRNA_7876, miR-100-5p, miR-148-3p	- 16 ncRNAs predicted PPCS with greater accuracy - 11 ncRNAs and age also identified symptom recovery (balance and cognitive test) from PPCS - Combining ncRNAs, balance, cognition identified recovery most accurately	Performance of 16 ncRNAs to predict PPCS AUC 0.86 (0.84–0.88)
Hicks, S. D. et al. (2018) [35]	Case–cohort	Children with sTBI and mTBI (salivary group and CSF group)	78 Salivary group *	60 mTBI	18 controls	29 F 31 M	5–21 y	Within 14 days after injury	NGS	miR-182-5p, miR-221-3p, miR-26b-5p, miR-320c, miR-29c-3p, miR-30e5p	- Six salivary miRNAs showed accurate potential to diagnose mTBI. These miRNAs also reflect the CSF patterns in sTBI.	The performance of 6 miRNAs to diagnose mTBI was: AUC = 0.852 (0.69–0.98) with similar validation accuracy (AUC 0.8)
Hicks, S. D. et al. (2020) [36]	Case- control	Former football athletes with diagnosed or recurrent concussion	31	13 participants	18 (age and sex matched)	31 M	46–89 y	8–18 h to collect form all participants	NGS	miR-101-3p, miR-582-3p, miR-424-5p, miR-340-5p, miR-181c-5p, miR-155-5p, miR-28-3p, miR-26b-5p, miR-30a-3p, miR-3184-3p, miR-423-5p, miR-4776-5p, miR-339-3p, miR-576-5p, miR-361-5p, miR-3074-5p, miR-24-3p, miR-574-5p, miR23a-3p, miR-23b-3p	- 20 salivary miRNAs show difference in expression between cases and controls - 9 miRNAs were reduced in football athletes, while 11 miRNAs were elevated - 2 of the miRNAs (miR-28-3p and miR-339-3p) showed association with number of prior concussions reported	NA
	Cross sectional	NA	310	230 without history of concussion	80 with single or recurrent concussion	102 F 208 M	7–39 y	7–19 h to collect from all participants				
Hicks, S. D. et al. (2020) [38]	Case-control	Patients with mTBI and controls	538 participants	251 mTBI (201 mTBI cases used for testing)	287 controls (no mTBI in the last 12 weeks) (229 controls used for testing)	207 F 331 M	5–66 Mean (SD) 18 (±6) y	≤14 days postinjury Saliva sample collected 5 timepoints post injury	NGS	miR-4510, miR-34a-5p, miR-744-5p, miR-192-5p, miR-25-3p, miR-30e-3p, miR-30a-3p, miR-3074-5p, miR-3614-5p, miR-378a-5p, miR-27a-5p, miR-181c-5p, miR-708-5p, miR-1246, let-7e-5p, miR-944, miR-1290, miR-181a-5p, miR-582-3p, miR-183-5p, miR-1180-3p, miR-12136	- A model containing 7 sncRNAs along with age and chronic headache was able to differentiate mTBIs from controls	Training group: AUC 0.857; (0.836-0.918)
			108 (Testing group)	50 mTBI for training	58 controls using for training							Testing group: AUC 0.823
Hicks, S. D. et al. (2021) [37]	Case-control	Athletes with SRC-related concussion and non-concussed athletes as comparator	172 participants with miRNAs not affected by exercise	75 concussed	97 non-concussed	198 M 116 F	8–58 y	≤24 h post-injury (for cases)	NGS	15 out of 40 miRNAs were unaffected by acute exercise and listed below: miR-27a-5p, miR-1246, miR-30e-3p, miR-30a-3p, miR-151a-3p, miR-192-5p, miR-7-1-3p, miR-181c-5p, miR-30e-5p, miR-1307-5p, miR-182-5p, miR-3074-5p, miR-629-5p, miR-944, miR-27b-3p	- 11 out of 15 miRNAs not confounded by exercise showed a significant difference between concussed and non-concussed participants. - The salivary miRNAs unaffected by exercise represent promising biomarker candidates for SRC	- Panel of 6 miRNAs displayed moderate ability (AUC > 0.70) to identify concussion. - A single ratio (miR-27a-5p/miR-30a-3p) displayed the highest accuracy (AUC = 0.810, sensitivity = 82.4%, specificity = 73.3%) for differentiating concussed and non-concussed participants. - miR-27a-5p was the most accurate miRNA for differentiating concussed and non-concussed participants - (AUC = 0.78, sensitivity = 74%, specificity = 75%).
Johnson, J. J. et al. (2018) [39]	Prospective cohort	mTBI patients with acute or prolonged symptoms	52 participants	30 with prolonged symptom (PCS) group	22 acute symptom (ACS) group	22 F 30 M	7–21 y Mean (SD) 14 (±3) y	14 days of injury	NGS	miR-769-5p, miR-4792, miR-629-5p, let-7a-5p, miR-320c-1, miR-140-3p, miR-133a-5p, let-7b-5p, miR-192-5p, miR-30e, miR-4508, miR-1307-3p, miR-200b-3p, miR-145-5p, miR-629	- 15 salivary miRNAs differentiated participants with PCS from ACS groups - Salivary miRNA levels may identify the duration and character of concussion symptoms	- A regression model using 5 miRNAs (miR-320c-1, miR-133a-5p, miR-769-5p, let-7a-3p, andmiR-1307-3p) demonstrated the highest diagnostic accuracy AUC, 0.856; 0.822-0.890)
												- Validation model demonstrated an AUC of 0.812; 0.691-0.893) for identification of PCS
												- Validation of the main model holding out 20% of random participant an AUC of 0.933 (0.787–0.960)
LaRocca, D. et al. (2019) [40]	Cohort study	Adult mixed martial arts fighters	42 MMA fighters	MMAs with HTH	Those who - had 0–3 HTH - their fight is cancelled on-site due to a reason	2 F 40 M	Mean (SD) 26.5 (±5.8) y	- 1 week or 1 h pre-fight - Within 15–30 min post-fight - 2–3 days, 1 week, and 3 weeks post-fight	NGS	miR-7-1-3p, miR-10a-5p, miR-10b-5p, miR-20a-5p, miR-30b-5p, miR-92a-3p, miR-122-5p, miR-128-3p, miR-155-5p, miR-455-5p, miR-1307-3p, miR-3146, miR-3678-3p miR-376a-5p, miR-4637, miR-4649-3p miR-4693-5p, miR-4766-5p, miR-5694, miR-6770-5p, miR-6809-3p	21 microRNAs from saliva and serum - microRNAs predicted mTBI accurately regardless of fluid type - Separate regression model containing 13 subsets of salivary miRNAs achieved perfect classification of mTBI	Salivary miRNAs combined with serum miRNAs provided AUC of 0.89 for predicting mTBI

Abbreviations: AUC, area under the curve; HTH, hits to head; MMA, mixed martial arts; NGS, next generation sequencing; ROC, receiver operating curve; SRC, sport related concussion.

**Table 2:** Prevalence and direction of change for miRNAs appearing in multiple studies.

miRNA	Di Pietro 2018 [33]	Di Pietro 2021 [32]	Fedorchak 2021 [34]	Hicks 2018 [35]	Hicks 2020 [36]	Hicks 2020 [38]	Hicks 2021 [37]	Johnson 2018 [39]	Larocca 2019 [40]
miR-1246		DOWN	DOWN		UP		DOWN		
miR-30a-3p					UP	DOWN	DOWN		
miR-181c-5p					UP	DOWN	DOWN		
miR-192-5p					UP		DOWN	UP	
miR-3074-5p					DOWN	UP	UP		
let-7a-5p		UP						DOWN	
let-7i-5p *	UP	UP							
miR-107 *	UP	UP							
miR-135b-5p *	UP	UP							
miR-148a-3p *	UP	UP							
miR-92a-3p		UP							DOWN
miR-20a-5p *	UP								UP
miR-24-3p *	UP					UP			
miR-27b-3p *	UP						UP		
miR-29c-3p *	UP			UP					
miR-181a-5p *	UP				UP				
miR-221-3p	UP			DOWN					
miR-424-5p	UP					DOWN			
miR-182-5p *				DOWN			DOWN		
miR-26b-5p *				DOWN		DOWN			
miR-320c *				DOWN				DOWN	
miR-30e-5p				UP			DOWN		
miR-582-3p					UP	DOWN			
miR-155-5p						DOWN			UP
miR-27a-5p *					DOWN		DOWN		
miR-30e-3p					UP		DOWN		
miR-7-1-3p *							DOWN		DOWN
miR-629-5p							UP	DOWN	
miR-944					UP		DOWN		
miR-1307-3p								UP	DOWN

Bolding and * indicate consistent directionality of the microRNA across all studies. microRNA upregulation is denoted by red cells, downregulation by blue cells.

## Data Availability

Not applicable.

## Supplementary Materials

The supporting information can be downloaded at: https://www.mdpi.com/article/10.3390/ijms232113160/s1

## References

[1] Khellaf A., Khan D.Z., Helmy A. (2019). Recent advances in traumatic brain injury. J. Neurol..

[2] Pan Y.B., Sun Z.L., Feng D.F. (2017). The Role of MicroRNA in Traumatic Brain Injury. Neuroscience.

[3] Dewan M.C., Rattani A., Gupta S., Baticulon R.E., Hung Y.-C., Punchak M., Agrawal A., Adeleye A.O., Shrime M.G., Rubiano A.M. (2018). Estimating the global incidence of traumatic brain injury. J. Neurosurg..

[4] Menon D.K., Schwab K., Wright D.W., Maas A.I. (2010). Position statement: Definition of traumatic brain injury. Arch. Phys. Med. Rehabil..

[5] Miller G.F., Daugherty J., Waltzman D., Sarmiento K. (2021). Predictors of traumatic brain injury morbidity and mortality: Examination of data from the national trauma data bank: Predictors of TBI morbidity & mortality. Injury.

[6] Rondina C., Videtta W., Petroni G., Lujan S., Schoon P., Mori L.B., Matkovich J., Carney N., Chesnut R. (2005). Mortality and morbidity from moderate to severe traumatic brain injury in Argentina. J. Head Trauma Rehabil..

[7] Harvey L.A., Close J.C. (2012). Traumatic brain injury in older adults: Characteristics, causes and consequences. Injury.

[8] Hiskens M., Vella R., Schneiders A., Fenning A. (2021). Celecoxib in a preclinical model of repetitive mild traumatic brain injury: Hippocampal learning deficits persist with inflammatory and excitotoxic neuroprotection. Trauma Care.

[9] Hiskens M.I. (2022). Targets of Neuroprotection and Review of Pharmacological Interventions in Traumatic Brain Injury. J. Pharmacol. Exp. Ther..

[10] McCrory P., Meeuwisse W., Dvorak J., Aubry M., Bailes J., Broglio S., Cantu R.C., Cassidy D., Echemendia R.J., Castellani R.J. (2017). Consensus statement on concussion in sport—The 5th international conference on concussion in sport held in Berlin, October 2016. Br. J. Sport. Med..

[11] Cota M.R., Moses A.D., Jikaria N.R., Bittner K.C., Diaz-Arrastia R.R., Latour L.L., Turtzo L.C. (2019). Discordance between Documented Criteria and Documented Diagnosis of Traumatic Brain Injury in the Emergency Department. J. Neurotrauma.

[12] Pin E., Petricoin E.F., Cortes N., Bowman T.G., Andersson E., Uhlen M., Nilsson P., Caswell S.V. (2021). Immunoglobulin A Autoreactivity toward Brain Enriched and Apoptosis-Regulating Proteins in Saliva of Athletes after Acute Concussion and Subconcussive Impacts. J. Neurotrauma.

[13] Bowman K., Matney C., Berwick D.M. (2022). Improving Traumatic Brain Injury Care and Research: A Report From the National Academies of Sciences, Engineering, and Medicine. JAMA.

[14] Huff J.S., Jahar S. (2014). Differences in interpretation of cranial computed tomography in ED traumatic brain injury patients by expert neuroradiologists. Am. J. Emerg. Med..

[15] Wilde E.A., Wanner I.-B., Kenney K., Gill J., Stone J.R., Disner S., Schnakers C., Meyer R., Prager E.M., Haas M. (2022). A Framework to Advance Biomarker Development in the Diagnosis, Outcome Prediction, and Treatment of Traumatic Brain Injury. J. Neurotrauma.

[16] Thelin E.P., Nelson D.W., Bellander B.M. (2017). A review of the clinical utility of serum S100B protein levels in the assessment of traumatic brain injury. Acta Neurochir..

[17] Hiskens M.I., Schneiders A.G., Angoa-Perez M., Vella R.K., Fenning A.S. (2020). Blood biomarkers for assessment of mild traumatic brain injury and chronic traumatic encephalopathy. Biomarkers.

[18] Zetterberg H., Blennow K. (2016). Fluid biomarkers for mild traumatic brain injury and related conditions. Nat. Rev. Neurol..

[19] Nguyen R., Fiest K.M., McChesney J., Kwon C.-S., Jette N., Frolkis A.D., Atta C., Mah S., Dhaliwal H., Reid A. (2016). The International Incidence of Traumatic Brain Injury: A Systematic Review and Meta-Analysis. The Canadian journal of neurological sciences Le journal canadien des sciences neurologiques. Can. J. Neurol. Sci..

[20] Kellermann I., Kleindienst A., Hore N., Buchfelder M., Brandner S. (2016). Early CSF and Serum S100B Concentrations for Outcome Prediction in Traumatic Brain Injury and Subarachnoid Hemorrhage. Clin. Neurol. Neurosurg..

[21] Hawkins T., Greenslade J.H., Suna J., Williams J., Rickard C.M., Jensen M., Donohue M., Cho E., Van Hise C., Egerton-Warburton D. (2018). Peripheral Intravenous Cannula Insertion and Use in the Emergency Department: An Intervention Study. Acad. Emerg. Med..

[22] Nam J.-W., Rissland O.S., Koppstein D., Abreu-Goodger C., Jan C.H., Agarwal V., Yildirim M.A., Rodriguez A., Bartel D.P. (2014). Global analyses of the effect of different cellular contexts on microRNA targeting. Mol. Cell.

[23] Redell J.B., Moore A.N., Ward N.H., Hergenroeder G.W., Dash P.K. (2010). Human traumatic brain injury alters plasma microRNA levels. J. Neurotrauma.

[24] Bartel D.P. (2004). MicroRNAs: Genomics, biogenesis, mechanism, and function. Cell.

[25] Di Pietro V., Ragusa M., Davies D., Su Z., Hazeldine J., Lazzarino G., Hill L.G., Crombie N., Foster M., Purrello M. (2017). MicroRNAs as novel biomarkers for the diagnosis and prognosis of mild and severe traumatic brain injury. J. Neurotrauma.

[26] Das Gupta S., Ciszek R., Heiskanen M., Lapinlampi N., Kukkonen J., Leinonen V., Puhakka N., Pitkänen A. (2021). Plasma miR-9-3p and miR-136-3p as Potential Novel Diagnostic Biomarkers for Experimental and Human Mild Traumatic Brain Injury. Int. J. Mol. Sci..

[27] Gilad S., Meiri E., Yogev Y., Benjamin S., Lebanony D., Yerushalmi N., Benjamin H., Kushnir M., Cholakh H., Melamed N. (2008). Serum microRNAs are promising novel biomarkers. PLoS ONE..

[28] Bhomia M., Balakathiresan N.S., Wang K.K., Papa L., Maheshwari R.K. (2016). A Panel of Serum MiRNA Biomarkers for the Diagnosis of Severe to Mild Traumatic Brain Injury in Humans. Sci. Rep..

[29] Mitra B., Rau T.F., Surendran N., Brennan J.H., Thaveenthiran P., Sorich E., Fitzgerald M.C., Rosenfeld J.V., Patel S.A. (2017). Plasma micro-RNA biomarkers for diagnosis and prognosis after traumatic brain injury: A pilot study. J. Clin. Neurosci..

[30] Methley A.M., Campbell S., Chew-Graham C., McNally R., Cheraghi-Sohi S. (2014). PICO, PICOS and SPIDER: A comparison study of specificity and sensitivity in three search tools for qualitative systematic reviews. BMC Health Serv. Res..

[31] Moher D., Liberati A., Tetzlaff J., Altman D.G., Group P. (2009). Preferred reporting items for systematic reviews and meta-analyses: The PRISMA statement. PLoS Med..

[32] Di Pietro V., O’Halloran P., Watson C.N., Begum G., Acharjee A., Yakoub K.M., Bentley C., Davies D.J., Iliceto P., Candilera G. (2021). Unique diagnostic signatures of concussion in the saliva of male athletes: The Study of Concussion in Rugby Union through MicroRNAs (SCRUM). BJSM.

[33] Di Pietro V., Porto E., Ragusa M., Barbagallo C., Davies D., Forcione M., Logan A., Pietro C.D., Purrello M., Grey M. (2018). Salivary MicroRNAs: Diagnostic Markers of Mild Traumatic Brain Injury in Contact-Sport. Front. Mol. Neurosci..

[34] Fedorchak G., Rangnekar A., Onks C., Loeffert A.C., Loeffert J., Olympia R.P., DeVita S., Leddy J., Haider M.N., Roberts A. (2021). Saliva RNA biomarkers predict concussion duration and detect symptom recovery: A comparison with balance and cognitive testing. J. Neurol..

[35] Hicks S.D., Johnson J., Carney M.C., Bramley H., Olympia R.P., Loeffert A.C., Thomas N.J. (2018). Overlapping MicroRNA Expression in Saliva and Cerebrospinal Fluid Accurately Identifies Pediatric Traumatic Brain Injury. J. Neurotrauma.

[36] Hicks S.D., Olympia R.P., Onks C., Kim R.Y., Zhen K.J., Fedorchak G., DeVita S., Rangnekar A., Heller M., Zwibel H. (2020). Saliva microRNA Biomarkers of Cumulative Concussion. Int. J. Mol. Sci..

[37] Hicks S.D., Onks C., Kim R.Y., Zhen K.J., Loeffert J., Loeffert A.C., Olympia R.P., Fedorchak G., DeVita S., Gagnon Z. (2021). Refinement of saliva microRNA biomarkers for sports-related concussion. J. Sport Health Sci..

[38] Hicks S.D., Onks C., Kim R.Y., Zhen K.J., Loeffert J., Loeffert A.C., Olympia R.P., Fedorchak G., DeVita S., Rangnekar A. (2020). Diagnosing mild traumatic brain injury using saliva RNA compared to cognitive and balance testing. Clin. Transl. Med..

[39] Johnson J.J., Loeffert A.C., Stokes J., Olympia R.P., Bramley H., Hicks S.D. (2018). Association of Salivary MicroRNA Changes With Prolonged Concussion Symptoms. JAMA Pediatr..

[40] LaRocca D., Barns S., Hicks S.D., Brindle A., Williams J., Uhlig R., Johnson P., Neville C., Middleton F.A. (2019). Comparison of serum and saliva miRNAs for identification and characterization of mTBI in adult mixed martial arts fighters. PLoS ONE.

[41] Zhou Q., Yin J., Wang Y., Zhuang X., He Z., Chen Z., Yang X. (2021). MicroRNAs as potential biomarkers for the diagnosis of Traumatic Brain Injury: A systematic review and meta-analysis. Int. J. Med. Sci..

[42] Chen J.J., Hsueh H., Delongchamp R.R., Lin C., Tsai C. (2007). Reproducibility of microarray data: A further analysis of microarray quality control (MAQC) data. BMC Bioinform..

[43] Novianti P.W., Roes K.C., Eijkemans M.J. (2014). Evaluation of gene expression classification studies: Factors associated with classification performance. PLoS ONE.

[44] Buschmann D., Haberberger A., Kirchner B., Spornraft M., Riedmaier I., Schelling G., Pfaffl M.W. (2016). Toward reliable biomarker signatures in the age of liquid biopsies-how to standardize the small RNA-Seq workflow. Nucleic Acids Res..

[45] Goutnik M., Lucke-Wold B. (2022). Commentary: Evaluating potential glioma serum biomarkers, with future applications. World J. Clin. Oncol..

[46] Schulte L.N., Eulalio A., Mollenkopf H.J., Reinhardt R., Vogel J. (2011). Analysis of the host microRNA response to Salmonella uncovers the control of major cytokines by the let-7 family. EMBO J..

[47] Balakathiresan N., Bhomia M., Chandran R., Chavko M., McCarron R.M., Maheshwari R.K. (2012). MicroRNA let-7i is a promising serum biomarker for blast-induced traumatic brain injury. J. Neurotrauma.

[48] Sabirzhanov B., Zhao Z., Stoica B.A., Loane D., Wu J., Borroto C., Dorsey S.G., Faden A.I. (2014). Downregulation of miR-23a and miR-27a following experimental traumatic brain injury induces neuronal cell death through activation of proapoptotic Bcl-2 proteins. J. Neurosci. Off. J. Soc. Neurosci..

[49] Chen Q., Xu J., Li L., Li H., Mao S., Zhang F., Zen K., Zhang C.-Y., Zhang Q. (2014). MicroRNA-23a/b and microRNA-27a/b suppress Apaf-1 protein and alleviate hypoxia-induced neuronal apoptosis. Cell Death Dis..

[50] Xi T., Jin F., Zhu Y., Wang J., Tang L., Wang Y., Liebeskind D.S., Scalzo F., He Z. (2018). miR-27a-3p protects against blood–brain barrier disruption and brain injury after intracerebral hemorrhage by targeting endothelial aquaporin-11. J. Biol. Chem..

[51] Li X.Q., Lv H.W., Wang Z.L., Tan W.F., Fang B., Ma H. (2015). MiR-27a ameliorates inflammatory damage to the blood-spinal cord barrier after spinal cord ischemia: Reperfusion injury in rats by downregulating TICAM-2 of the TLR4 signaling pathway. J. Neuroinflamm..

[52] Shultz S.R., Taylor C.J., Aggio-Bruce R., O’Brien W.T., Sun M., Cioanca A.V., Neocleous G., Symons G.F., Brady R.D., Hardikar A.A. (2022). Decrease in Plasma miR-27a and miR-221 After Concussion in Australian Football Players. Biomark. Insights.

[53] Savaskan N.E., Brauer A.U., Nitsch R. (2004). Molecular cloning and expression regulation of PRG-3, a new member of the plasticity-related gene family. Eur. J. Neurosci..

[54] Lopez J.P., Fiori L.M., Gross J.A., Labonte B., Yerko V., Mechawar N., Turecki G. (2014). Regulatory role of miRNAs in polyamine gene expression in the prefrontal cortex of depressed suicide completers. Int. J. Neuropsychopharmacol..

[55] Wang Y.T., Tsai P.C., Liao Y.C., Hsu C.Y., Juo S.H.H. (2013). Circulating microRNAs have a sex-specific association with metabolic syndrome. J. Biomed. Sci..

[56] Morgan C.P., Bale T.L. (2012). Sex differences in microRNA regulation of gene expression: No smoke, just miRs. Biol. Sex Differ..

[57] Rekker K., Saare M., Roost A.M., Salumets A., Peters M. (2013). Circulating microRNA Profile throughout the menstrual cycle. PLoS ONE.

[58] Luizon M.R., Conceição I.M.C.A., Viana-Mattioli S., Caldeira-Dias M., Cavalli R.C., Sandrim V.C. (2021). Circulating MicroRNAs in the Second Trimester From Pregnant Women Who Subsequently Developed Preeclampsia: Potential Candidates as Predictive Biomarkers and Pathway Analysis for Target Genes of miR-204-5p. Front. Physiol..

[59] Witwer K.W., Hirschi K.D. (2014). Transfer and functional consequences of dietary microRNAs in vertebrates: Concepts in search of corroboration: Negative results challenge the hypothesis that dietary xenomiRs cross the gut and regulate genes in ingesting vertebrates, but important questions persist. Bioessays.

[60] Gomes C.P., Oliveira G.P., Jr Madrid B., Almeida J.A., Franco O.L., Pereira R.W. (2014). Circulating miR-1, miR-133a, and miR-206 levels are increased after a half-marathon run. Biomarkers.

[61] Nielsen S., Åkerström T., Rinnov A.R., Yfanti C., Scheele C., Pedersen B.K., Laye M.J. (2014). The miRNA plasma signature in response to acute aerobic exercise and endurance training. PLoS ONE.

[62] Horak M., Zlamal F., Iliev R., Kučera J., Cacek J., Svobodova L., Hlavoňová Z., Kalina T., Slaby O., Bienertova-Vasku J. (2018). Exercise-induced circulating microRNA changes in athletes in various training scenarios. PLoS ONE.

[63] Eyileten C., Wicik Z., Fitas A., Marszalek M., Simon J.E., De Rosa S., Wiecha S., Palatini J., Postula M., Malek L.A. (2021). Altered Circulating MicroRNA Profiles After Endurance Training: A Cohort Study of Ultramarathon Runners. Front. Physiol..

[64] Uhlemann M., Möbius-Winkler S., Fikenzer S., Adam J., Redlich M., Möhlenkamp S., Hilberg T., Schuler G.C., Adams V. (2014). Circulating microRNA-126 increases after different forms of endurance exercise in healthy adults. Eur. J. Prev. Cardiol..

[65] Pritchard C.C., Kroh E., Wood B., Arroyo J.D., Dougherty K.J., Miyaji M.M., Tait J.F., Tewari M. (2012). Blood Cell Origin of Circulating MicroRNAs: A Cautionary Note for Cancer Biomarker StudiesCirculating MicroRNA Biomarkers and Blood Cells. Cancer Prev. Res..

[66] Majem B., Rigau M., Reventós J., Wong D.T. (2015). Non-coding RNAs in saliva: Emerging biomarkers for molecular diagnostics. Int. J. Mol. Sci..

[67] Sullan M.J., Asken B.M., Jaffee M.S., DeKosky S.T., Bauer R.M. (2018). Glymphatic system disruption as a mediator of brain trauma and chronic traumatic encephalopathy. Neurosci. Biobehav. Rev..

[68] Sun P., Liu D.Z., Jickling G.C., Sharp F.R., Yin K.J. (2018). MicroRNA-based therapeutics in central nervous system injuries. J. Cereb. Blood Flow Metab..

[69] Madathil S.K., Nelson P.T., Saatman K.E., Wilfred B.R. (2011). MicroRNAs in CNS injury: Potential roles and therapeutic implications. Bioessays.

[70] Hiskens M.I., Vella R.K., Schneiders A.G., Fenning A.S. (2021). Minocycline improves cognition and molecular measures of inflammation and neurodegeneration following repetitive mTBI. Brain Inj..

