# Supplementary material for: Systematic Review of the Diagnostic and Clinical Utility of Salivary microRNAs in Traumatic Brain Injury (TBI)
Source: Int J Mol Sci. 2022 Oct 29;23(21):13160. doi: 10.3390/ijms232113160 (PMC9654991; doi:10.3390/ijms232113160)
Supplement: Supplementary file 1 [file ijms-23-13160-s001.zip › ijms-1952822-supplementary.pdf]

Supplementary Table S1. Databases and search strategies

Data base	Search strategies	Number of citations retrieved
EMBASE	('microrna'/exp OR 'mirna':ti,ab,kw OR 'mirnas':ti,ab,kw OR 'micro rna':ti,ab,kw OR 'microrna':ti,ab,kw OR 'micrornas':ti,ab,kw OR biomsrker:ti,ab OR 'saliva'/exp OR 'saliva':ti,ab,kw OR 'spittle':ti,ab,kw) AND ('traumatic brain injury'/exp OR 'brain injuries, traumatic':ti,ab,kw OR 'brain lesion, traumatic':ti,ab,kw OR 'brain system trauma':ti,ab,kw OR 'brain trauma':ti,ab,kw OR 'cerebral trauma':ti,ab,kw OR 'cerebrovascular trauma':ti,ab,kw OR 'encephalopathy, traumatic':ti,ab,kw OR 'mild traumatic brain injury':ti,ab,kw OR 'organic cerebral trauma':ti,ab,kw OR 'posttraumatic encephalopathy':ti,ab,kw OR 'traumatic brain injuries':ti,ab,kw OR 'traumatic brain injury':ti,ab,kw OR 'traumatic brain lesion':ti,ab,kw OR 'traumatic cerebral lesion':ti,ab,kw OR 'traumatic encephalopathy':ti,ab,kw OR tbi:ti,ab OR 'chronic traumatic encephalopathy'/exp OR 'chronic traumatic brain injury':ti,ab,kw OR 'chronic traumatic encephalopathy':ti,ab,kw OR 'dementia pugulistica':ti,ab,kw OR cte:ti,ab) AND [humans]/lim AND [english]/lim AND [clinical study]/lim	156
Scopus	(TITLE-ABS (microRNA OR mirRNA OR miRNAs OR "micro RNA" OR microRNAs OR biomarker* OR saliva)) AND (TITLE-ABS (diagnos* OR prognos*)) AND (TITLE-ABS("traumatic brain injury" OR tbi OR "brain injur*" OR "traumatic brain lesion" OR "brain system trauma" OR "brain trauma" OR "cerebral trauma" OR "cerebrovascular trauma" OR "traumatic encephalopathy" OR "mild traumatic brain injury" OR mtbi OR "organic cerebral trauma")) AND (LIMIT-TO (SRCTYPE,"j")) AND (LIMIT-TO (PUBSTAGE,"final")) AND (LIMIT-TO (DOCTYPE,"ar")) AND (LIMIT-TO (LANGUAGE,"English")) AND (LIMIT-TO (EXACTKEYWORD,"Human") OR LIMIT-TO (EXACTKEYWORD,"Humans")) AND (LIMIT-TO (SUBJAREA,"MEDI") OR LIMIT-TO (SUBJAREA,"NEUR") OR LIMIT-TO (SUBJAREA,"NURS"))	580
PsycINFO	((((title: (microrna)) OR (title: (mirna)) OR (title: (mirnas)) OR (title: ("micro rna")) OR (title: (micrornas)) OR (title: (biomarker*)) OR (title: (saliva)))) AND (title: (diagnos*))) OR (title: (prognos*))) OR (title: (predict*)))) AND ((((((MeSH: (traumatic brain injury))) OR ((MeSH: (tbi))) OR ((MeSH: (brain injur*))) OR ((MeSH: (traumatic brain lesion))) OR ((MeSH: (brain system trauma))) OR ((MeSH: (brain trauma))) OR ((MeSH: (cerebral trauma))) OR ((MeSH: (cerebrovascular trauma))) OR ((MeSH: (traumatic encephalopathy))) OR ((MeSH: (mild traumatic brain injury))) OR ((MeSH: (mtbi))) OR ((MeSH: (organic cerebral trauma))))))) AND Population Group: Human AND Document Type: Journal Article	484
Web of Science	(ALL=(microrna OR mirna OR mirnas OR micro rna OR microrna OR micrornas OR biomsrker* OR saliva) AND ALL=((diagnos* OR prognos* OR predict*)) AND ALL=(traumatic brain injury OR TBI OR brain injur* OR brain lesion OR brain system trauma OR brain trauma OR cerebral trauma OR cerebrovascular trauma OR traumatic encephalopathy OR mild traumatic brain injury OR mtbi OR organic cerebral trauma OR posttraumatic encephalopathy OR traumatic brain lesion OR traumatic cerebral lesion OR traumatic encephalopathy OR CTE OR chronic traumatic encephalopathy)) AND (DT==("ARTICLE") AND LA==("ENGLISH") AND DT==("ARTICLE") AND OA==("OPEN ACCESS"))	358
PubMed	(((("microrna"[tiab] OR "mirna"[tiab] OR "mirnas"[tiab] OR "micro rna"[tiab] OR "micrornas"[tiab] OR "biomarker*"[tiab] OR "saliva"[tiab]) AND "diagnos*"[tiab]) OR "prognos*"[tiab] OR "predict*"[tiab]) AND ("brain injuries, traumatic"[MeSH Terms] OR ("brain"[All Fields] AND "injuries"[All Fields] AND "traumatic"[All Fields]) OR "traumatic brain injuries"[All Fields] OR ("traumatic"[All Fields] AND "brain"[All Fields] AND "injury"[All Fields]) OR "traumatic brain injury"[All Fields] OR "traumatic brain injury"[tiab] OR "TBI"[tiab] OR "traumatic brain injuries"[tiab] OR "brain trauma"[tiab] OR "cerebral trauma"[tiab] OR "cerebrovascular trauma"[tiab] OR "encephalopathy traumatic"[tiab] OR "mild traumatic brain injury"[tiab] OR "posttraumatic encephalopathy"[tiab] OR "traumatic brain injuries"[tiab] OR "traumatic brain lesion"[tiab] OR "traumatic cerebral lesion"[tiab] OR "traumatic encephalopathy"[tiab] OR "CTE"[tiab] OR "chronic traumatic encephalopathy"[tiab])) AND ((ffrft[Filter]) AND (clinicalstudy[Filter] OR clinicaltrial[Filter] OR comparativestudy[Filter] OR controlledclinicaltrial[Filter] OR multicenterstudy[Filter] OR observationalstudy[Filter] OR randomizedcontrolledtrial[Filter] OR validationstudy[Filter]) AND (humans[Filter]) AND (english[Filter]))	552
